# Correction to ‘Knowledge and Attitudes of Palliative Care Among Community Health Nurses in China: A Cross‐Sectional Study’

**DOI:** 10.1002/nop2.70445

**Published:** 2026-02-09

Liu, D. X., L. N. Kong, J. Yang, and Q. Lyu. 2025. “Knowledge and Attitudes of Palliative Care Among Community Health Nurses in China: A Cross‐Sectional Study.” Nursing Open 12, no. 12:e70389. https://doi.org/10.1002/nop2.70389.

The article type ‘DISCURSIVE PAPER’ was incorrect. This should have been ‘Empirical Research Quantitative’.

We apologise for this error.

